# Beyond the business-to-client model: how the business-to-business model can transform the complex patient care

**DOI:** 10.3325/cmj.2024.65.400

**Published:** 2024-08

**Authors:** Dorja Vočanec, Aleksandar Džakula, Karmen Lončarek

**Affiliations:** 1 Department of Social Medicine and Organization of Health Care, Andrija Štampar School of Public Health, University of Zagreb School of Medicine, Zagreb, Croatia; *dvocanec@snz.hr*; 2 Center for Integrated and Palliative Care, University of Rijeka, Faculty of Medicine, Rijeka, Croatia

## From one-man band to pit crew in health care

If there is an adjective that precisely describes contemporary medicine and health care, it is “complex.” The explosive development of medical technologies and the possibilities of diagnosis and treatment have transformed what were once simple interventions for patients into the most complex biotechnological, ethical, and organizational challenges (1).

Even in simple linear examples, such as when someone falls in the street and breaks a hip, their care will involve an emergency medical services (EMS) dispatcher; an EMS doctor and medical technician in the vehicle; then a nurse and doctor at the emergency department; a radiology technician and radiologist; a medical laboratory technician and a medical biochemistry engineer; a surgeon, anesthesiologist, surgical and anesthesiology nurse, and a surgical technologist in the surgical department or operating room; a physiatrist and physiotherapist in the department. Additionally, auxiliary workers and associates who come into direct contact with the patient, such as a caregiver, porter, receptionist, and EMS driver, also play a role. This amounts to 16 professionals and four non-health care workers. If we included all the experts involved in rehabilitation and post-hospital care, the number would double.

## Patient-centered care models prove inadequate as the fragmentation of care increases

Such a modern leap in technologies challenges the position and role of the patient in health care. This challenge has been recognized in multiple dimensions, leading to attempts to adequately respond by developing various models of person-centered medicine, patient-centered health care, or even community-centered health care. However, despite the focus on the patient, with the mission, intention, and principles aimed at protecting the patient's role and position, it has not yielded results. The limited effectiveness of this model has become particularly evident with the emergence of an increasing number of complex patients. It was expected that with complex patients, we would see how successfully we have addressed the detachment of medicine from the human element and how important it is for the patient to be at the center of attention, especially regarding care outcomes, quality of life, and patient satisfaction. From the perspective of the traditional approach to health care, we were doing everything in the best way possible. There were those who provided services and care and those who received them. Translating this into the language of the business world, we had a well-established business-to-client (B2C) model. Although the number of professionals involved in care has multiplied, and the dynamics of their involvement in the care process have become more complicated (especially pronounced in complex patients in long-term care), the paradigm of the traditional doctor-patient relationship has persisted. This paradigm originated from a time when a patient visited one doctor for all their troubles, and that doctor provided care within the limits of what they independently knew and could do (2).

While medicine was increasingly trying to develop a B2C model by promoting the importance of the patient's role, the business world developed an entirely new concept: business-to-business (B2B).

## Patient as a bottleneck in professional collaboration

Complex patients often cannot actively participate in their treatment and rehabilitation as they have needs that mutually reinforce and worsen each other. Their caregivers are often strongly tied to them, which reduces the caregivers’ capacity to contribute to the organization and coordination of patient care. In the classic patient-centered health care system, the physician's focus on the patient narrows so much that it places the patient as an intermediary between the physician and other professionals who are necessarily involved. Thus, the physician communicates and delivers to the patient not only the information intended primarily for the patient but also the information meant for other professionals who should act together and in coordination.

Even in the description of this concept, patients and their caregivers become a bottleneck and cannot actively participate in the complex systems of modern health care. Additionally, this approach is burdensome for the patient and unreliable as a method of information transfer, as some information gets lost or delayed while the patient functions as a messenger conveying verbal messages or a courier transporting written messages in the form of medical documents.

Other attempts for health care professionals to collaborate and communicate in patient care have often been reduced to an administrative process – a referral that must be present somewhere in the system to initiate the procedure administratively or a report on what has been done.

## The ambiguity of the B2B approach in health care

Therefore, various stakeholders involved in the care of complex patients should communicate and coordinate their activities directly and in real time. Unfortunately, years of fragmentation in modern health care have confined stakeholders into different professional silos, making their communication difficult, insufficient, or nonexistent. Each stakeholder will claim that what they have provided to the patient within their domain is the maximum possible under the given circumstances. These circumstances, based on the B2C model, prevent the networking of stakeholders and their direct collaboration. Care for complex patients clearly shows that the B2C model cannot meet the challenges of modern medicine and the needs of complex patients. This model exacerbates fragmentation and raises new barriers between stakeholders in the system, despite being declared as a model directly aimed at the patient's well-being (3-5).

Observing all the services received by complex patients as part of comprehensive care reveals a whole range of interactions between stakeholders who connect through service provision. These interactions include the exchange of diagnostic findings, information sharing, coordination of individual services, planning of interventions that need to be interconnected or coordinated, etc. Such an ecosystem created around the complex patient consists of professional collaboration, for which the B2C model does not yield results (6).

The numerous business interactions carried out by stakeholders in the health care system while caring for the same patient are actually a perfect representation of the B2B model in health care but follow a flawed logic. Additionally, many professionals consider their relationship with the patient as the foundation of their work (as they have been trained and educated this way), and view their primary job as treating patients. Consequently, they do not see relationships with other professionals as an essential part of their work, but rather as an incidental, unpleasant administrative obligation that disrupts their primary relationship with the patient. This lack of awareness about the need for both models of work among professionals results in an inadequate development of professional collaborations and causes significant frustration.

Parallel to this, the B2B model in the health care system has mostly been applied at the level of stakeholders involved in various segments that support the health care system. This includes technical support for the health care system, IT networking, suppliers, product delivery, quality control, and financial interventions. In other words, all forms of classic business services aimed at the health care sector or health care institutions. In this model, there has been no place for patients (7).

The existing B2B models in health care are oriented toward that segment of business where health care is represented by institutions or where there is a clear financial interest, ie, a flow of money.

## The necessary switch

If we want further development and integration of health care, health care professionals should be educated to build business relationships with other professionals as an integral part of their professional routine, in addition to their relationship with the patient. This includes a long-term development of business relationships, coordination and integration of services and care, and participation in decision-making, which consequently involves a different type of communication, language, content, and means. The subject of this communication does not necessarily pertain only to individual patients but also to other business elements such as business processes.

We are talking about a higher level of B2B model application, which implies a paradigm shift compared with traditional health care. We need a dual B2C and B2B model in health care, similar to what exists in large technology companies or taxi service companies.

This would shift the focus from individual service provision to integration and collaboration among health care providers. For example, hospitals, specialist clinics, laboratories, and medical equipment suppliers could form networks or partnerships that would facilitate information exchange, shared resource use, and coordinated care. This would also reduce the fragmentation of care that often creates barriers to effective treatment of complex cases.

Given that complex patients are already at the center of the network of health care interactions, recognizing and formalizing existing interactions as B2B relationships can help reduce operational challenges and improve treatment outcomes. Creating such collaborative networks could also enable better standardization of procedures and protocols, greater consistency in care, and better response to patient needs.
